# Observations on the Therapeutic Action of the Oxalate of Cerium in the Vomiting of Pregnancy

**Published:** 1861-02

**Authors:** H. W. Jones


### CHICAGO

### MEDICAL JOURNAL.

VOL. IV.] FEBRUARY, 1861. [NO. 2.

### ORIGINAL COMMUNICATIONS.

### OBSERVATIONS ON THE THERAPEUTIC ACTION OF THE OXALATE OF CERIUM IN THE VOMITING OF PREGNANCY.

BY H. W. JONES, A. M., M. D.

A BRIEF account of a series of experiments with the salts of Cerium, was presented to the medical profession, by Dr. Simpson, of Edinburgh, as early as November, 1854.

But I have seen no notice of the therapeutical action of this metal upon the economy, as observed by American physicians until quite recently.

In the October number of the American Journal of Medical Sciences, Dr. Charles Lee reports several interesting cases of nausea and vomiting, dyspeptic, or accompanying pregnancy, in which the happiest effects followed the administration of Cerium.

The results of my own experience, though meager, may serve at least to stimulate inquiry in a direction where there is, confessedly, much to be gained, both by physician and patient.

The following cases, from notes in obstetric practice, comprehend the several instances in which I have used the Oxalate of Cerium since August, 1860:

CASE FIRST.

Mrs. M. H., aged 26, consulted me August 3rd, 1860. She is of sanguine temperament, nervous, haggard in appearance. Says she is in the third month of her pregnancy, and has been subject to distressing attacks of nausea and vomiting since the disappearance of her menses. At first, these attacks were confined to the early hours of morning; but of late, she complains of being never entirely free from nausea, and that vomiting follows any attempt at deglutition, whether of solids or fluids.

Has taken alkalies and used counter-irritation in vain. The bowels are costive—tongue furred—pulse small and frequent; tenderness of epigastrium on pressure. Has general appearance of great prostration.

*Treatment.*—The bowels were moved by injections of cool water, and enemata of beef-tea, containing *Quinia*, were administered four times a day, with the addition of two grains of *Opium* at night. Ice in small quantities to be swallowed often during the day. No food was allowed by the mouth. Sinapisms over stomach, so as to afford continuous irritation, but not to vesicate.

August 5th—Patient has slept well—" feels stronger "—no tenderness on pressure over epigastrium; has taken and retained, this noon, a teaspoonful of milk porridge.

She continued in about the same condition for several days, nauseated and distressed in the morning, but able to bear a little milk or rice in the afternoon, in the meantime using small doses of *Hydrocyanic Acid*, with the mildest bitter tonics, which were in turn supplanted by other remedies, as they failed to afford relief.

Aug. 12th—The patient begins to-day upon the following:

℞ Cerii Oxalat, Gr. xxiv;
Sacchar. Alb. ʒ ss;
M. ft. chartul, No. xii.
S. To take one powder every three hours.

Aug. 13th—Had less nausea this morning; has taken a small portion of chicken with her rice this afternoon. "Feels hungry," but "fears to eat."

Continues the remedy once in four hours.

Aug. 15th—Patient had no nausea this morning; ate a small piece of steak for breakfast; looks much better.

From this time, her improvement was steady; the gastric irritability recurring slightly at quickening, but yielding speedily to the Cerium.

At present, (8th month,) she is "perfectly well," having not only a good appetite, but excellent digestion.

CASE SECOND.

Sept. 10th—Mrs. C., aged 32; bilious temperament; pregnant with her third child—in eighth month. Complains of "morning sickness;" sometimes severe vomiting and retching, with great prostration and attendant pain in back and loins; fears "miscarriage." Was similarly affected in the early weeks of this, but did not suffer much in her previous pregnancies. Bowels regular and habits good.

*Treatment.*—From its success in the former instance, the *Oxalate of Cerium* was at once prescribed, in doses of two grains, to be taken half an hour before rising from bed, and if necessary before each meal.

Sept. 24th—Patient has experienced but one attack of vomiting since I saw her, and that followed an omission of the remedy before breakfast. She was soon able to dispense with it altogether, and was delivered at term, having had no relapse.

CASE THIRD.

Oct. 28th—Mrs. R., aged 25; sanguine temperament; third pregnancy. Has, in former pregnancies, suffered so much from nausea and vomiting, as to render her incapable of attending to household duties, and deter her from the enjoyments of social life. Never found any relief from remedies advised. Does not know how far advanced she may be, as conception took place during lactation. Vomits usually before breakfast, and food at any time produces discomfort. System otherwise in good condition.

*Treatment.*—As in Case 2nd, with "entire relief" of the symptoms by the third day.

No return of the difficulty as yet. (Jan. 1st.)

CASE FOURTH.

Oct. 31st—Mrs. ——, aged 22—sanguine temperament; primipara at eighth month. Complains chiefly of want of appetite. Relishes nothing but pickles and spiced food, but is nauseated and griped after taking it. From third to sixteenth week suffered regularly from "morning sickness." Coffee then relieved her; now has no effect.

*Treatment.*—*Oxalate of Cerium*, two grains, three times a day, an hour before meals. Regulated diet: no acids allowed.

The appetite became better; unnatural cravings disappeared. Remedy given up after fifth day. Nausea sometimes recurs, but is speedily relieved by the powders.

CASE FIFTH.

Nov. 14th—Mrs. S., aged 20—nervous temperament; anæmic appearance. Was married at sixteen, and has had two abortions, one at second month, and the last, in third month, occurred last April. "Didn't know cause." First indication of present pregnancy, was increased frequency of micturition about middle of October. This subsided, and nausea, with vomiting, occurred in the first week of November, when she should have been "unwell." This symptom appeared also in previous pregnancies. Bowels are costive; has a dull and persistent headache—no appetite, and feels "very weak."

*Treatment.—Citrate of Magnesia*, pro re nata, with *Citrate of Iron* and *Quinia* in *Madeira Wine.*

Nov. 18th—Bowels soluble, headache gone, appetite better, but much nausea in morning. Tonics continued. *Oxalate of Cerium* advised night and morning, in doses of two grains, before meals.

Nov. 24th—General health much better; does not vomit, though nausea is present in morning as before. The Cerium was now taken every three hours, but proved inefficient, and was given up after a few days' trial. The case is progressing favorably, however, and I hope to carry it through to term. Shall recur to the Cerium if vomiting should again come on.

---

In these cases, I endeavored to isolate the remedy as far as possible, in order to avoid any misconception which might otherwise have arisen, as to what the amelioration of symptoms might be due, and I do not hesitate to give my confidence to the "*post hoc, propter hoc*" principle, as exhibited in the treatment and results above cited.

Dr. Lee has witnessed beneficial results from the *Cerium* in cases of vomiting in phthisis pyrosis—hysterical emesis—and in atonic dyspepsia, and it is to be hoped that further experiments will establish the usefulness of the remedy.

Dr. Simpson considers it a *sedative tonic*, and compares it favorably with the salts of *Bismuth*, advising its administration in all cases where the latter would be indicated.

"The *Oxalate of Cerium* is inodorous, tasteless, insoluble in water, alcohol or ether, but freely soluble in sulphuric acid, by which it may be distinguished from the other salts of the earth."

It is, I thing, best administered in the form of powder, though a pill form may be convenient. Two grains is a medium dose.

